# Notification of HIV status disclosure and its related factors in HIV-infected adolescents in 2009 in the Aconda program (CePReF, CHU Yopougon) in Abidjan, Côte d'Ivoire, The PRADO-CI Study

**DOI:** 10.7448/IAS.16.1.18569

**Published:** 2013-06-18

**Authors:** Guanga David Meless, Hortense Aka-Dago-Akribi, Chantal Cacou, Tanoh François Eboua, Addi Edmond Aka, Aimé Maxime Oga, Belinda Bouah, Messou Eugène, Corinne Moh, Elise Arrivé, Marguerite Timité-Konan, Valériane Leroy, for the PRADO-CI Study Group

**Affiliations:** 1 Programme PACCI, Abidjan, Côte d'Ivoire; 2 Département de Psychologie, Université de Cocody, Abidjan, Côte d'Ivoire; 3 Service de Pédiatrie, CHU de Yopougon, Abidjan, Côte d'Ivoire; 4 Centre de Prise en Charge, de Recherche et de Formation, CePReF, Aconda, Abidjan, Côte d'Ivoire; 5 Inserm, U897, Institute of Public Health, Université Bordeaux Segalen, Bordeaux, France; 6 Institute of Public Health, Université Bordeaux Segalen, Bordeaux, France

**Keywords:** adolescents and youth, sub-Saharan Africa, antiretroviral treatment, disclosure, HIV, prevention

## Abstract

**Introduction:**

We studied the frequency of documentation of disclosure of HIV status in medical charts and its correlates among HIV-infected adolescents in 2009, in Abidjan, Côte d'Ivoire.

**Methods:**

The PRADO-CI is a cross-sectional study aimed at studying HIV-infected adolescents’ social, psychological, and behavioural difficulties and their determinants in Abidjan, Côte d'Ivoire. In this study, we present specific analyses on disclosure. All HIV-infected adolescents aged 13–21 years and followed at least once in 2009 in two urban HIV-care centres in Abidjan (Cepref and Yopougon Teaching Hospital) were enrolled in the study. Standardized data were extracted from medical records to document if there was notification of disclosure of HIV status in the medical record. Frequency of notification of HIV disclosure was estimated with its 95% confidence interval (CI) and correlates were analyzed using logistic regression.

**Results:**

In 2009, 229 adolescents were included: 126 (55%) males; 93% on antiretroviral therapy (ART), 61% on cotrimoxazole prophylaxis. Their median age was 15 years at the time of the study. Among the 193 patients for whom information on HIV status disclosure was documented (84%), only 63 (32.6%; 95% CI=26.0–39.3%) were informed of their status. The proportion of adolescents informed increased significantly with age: 19% for 13–15 years, 33% for 16–18 years and 86% for 19–21 years (*p* <0.0001). Adolescents on ART tended to be more likely to be informed of their HIV status (34.5%) than those not treated (13.3%) (*p=*0.11). Those on cotrimoxazole were significantly more likely to be informed (39.6%) than those not (21.9%) (*p=*0.01). Disclosure was significantly higher in adolescents with a history of ART regimen change (*p=*0.003) and in those followed in the Cepref (48.4%) compared to the Yopougon Teaching Hospital (24.8%), (*p=*0.001). In multivariate analyses, disclosed HIV status was significantly higher in those followed-up in the Cepref compared to the other centre: adjusted odds ratio (aOR)=3.5 (95% CI: 1.1–10.9), and among older adolescents compared to those aged 13–15 years: [16–18 years] aOR=4.2 (95% CI: 1.5–11.5) and [>18 years]: aOR=22.1 (95% CI: 5.2–93.5).

**Conclusions:**

HIV disclosure rate was low among Ivoirian HIV adolescents and was site- and age-dependent. There is a need for practical interventions to support HIV disclosure to adolescents which provides age-appropriate information about the disease.

## Introduction

Worldwide, about 400,000 infants are infected with HIV through mother-to-child transmission (MTCT) annually [1]. These HIV-infected children live longer because of greater access to antiretroviral therapy (ART) and reach adolescence. This is an emerging and growing population with specific features as observed in Africa over the past decade [2, 3].

Adolescence is a crucial period of transition to adulthood, characterized by physical, mental, and social changes and challenges. Adolescents starting sexual activity are at high risk of HIV acquisition and transmission in areas of generalized epidemic [4–6]. HIV-infected adolescents face numerous challenges in coping with their disease, with mental health problems, and emotional and behavioural disorders, such as anxiety, depression, somatization or suicide attempts [7, 8]. Poor virological response to treatment may be associated with non-adherence and sub-optimal antiretroviral use [9]. Adherence to ART is a key issue for therapeutic long-term success. Poor adherence has been reported in both American and South African adolescents [10, 11].

Thus, as the number of young HIV-infected people increases, it becomes necessary to develop programmes offering age-appropriate care and treatment, providing psychosocial support, counselling on reproductive health and advocacy on their behalf. Therefore, it is crucial to understand that these problems cannot be adequately addressed in adolescents not informed of their HIV status. In an American context, children and adolescents who knew their HIV status appeared more likely to accept medical care and have a higher self-esteem compared to youth that were unaware of their status [12]. In resource-limited settings, disclosure of HIV status was identified as a factor strongly associated with better adherence [13] and higher retention in care [14].

Therefore, the World Health Organization (WHO) recommends national policies to implement programmes with tools and resources providing clear, specific guidance on disclosure of HIV [15]. However, the process and the resources available for training providers about paediatric HIV disclosure are largely based on the Western disclosure model and experience [16, 17] and are often not adapted for low-income countries. In addition, the disclosure process is not well described in sub-Saharan Africa. The prevalence of disclosure in children and adolescents varies according to methods, settings and age of the patients, but is generally low: between 1.7% and 38% in children between five and 17 years in sub-Saharan Africa [6, 8, 14, 18–31]. More specifically, in Zambia, the disclosure rate reached 38% among 127 adolescents aged between 11 and 15 years [8] and was 27% among 96 children at a median age of six years [13]. In Uganda, disclosure of HIV status by caregivers occurred in 29% of 42 children at a median age of 12 years [22]. In Ethiopia, the disclosure rate was only 17% among 390 children with a mean age of 8.5 years [21]. In South Africa, only 9% out of 174 caregivers had disclosed their status to their HIV-children aged 5–11 years [32]. In the Democratic Republic of Congo, only 3% of caregivers had informed their child of their HIV status among 259 children aged 5–17 years [29]. Finally, in Kenya, among 120 children of a mean aged 6.8 years, 1.7% were informed of their HIV status [31].

There are only three studies in west Africa, where HIV prevalence is lower. In Ghana, in a cross-sectional study of 71 caregiver-child pairs, the prevalence of disclosure to their HIV-infected children where children had a median age of 10 years was 21% [27]. Among 650 HIV-infected children aged 10 years or more in Côte d'Ivoire, Mali and Senegal, only 28.8% knew their HIV status [14]. In Nigeria, the caregivers of 96 HIV-infected children of a median age of 8.8 years reported disclosure in only 13.5% of the children [23]. To document what is known by healthcare workers about disclosure of HIV status in routine care, we studied the prevalence and characteristics of HIV disclosure status among HIV-infected adolescents in Abidjan, Côte d'Ivoire.

## Methods

### Settings

Since 2004, the Aconda antiretroviral programme has offered free care and ART to children in Côte d'Ivoire [33, 34]. This program was funded by the United States President's Emergency Plan for AIDS Relief (PEPFAR), through the Elizabeth Glaser Paediatric AIDS Foundation (EGPAF; Washington DC, USA) and supported by the French “Groupement d'Intêret Publique” (GIP) Esther. In Abidjan, the Aconda team trained health workers in HIV care and implemented a standardized computerized data management system in partnership with the Bordeaux School of Public Health (ISPED, France). Overall, in 2009, in the two main Abidjan paediatric centres, 2244 HIV-infected children (0–21 years) were followed in the Aconda active file, of which 1000 were on ART [33].

### Paediatric HIV care

The Aconda paediatric HIV care package includes systematic paediatric HIV diagnostic tests. In children over 18 months, the standard serologic testing algorithm was a series of two rapid HIV assays: Determine^®^ HIV-1/2 (Abbott Diagnostics, Abbott Park, IL, USA), followed by Geni II^®^ HIV1/HIV2 (Bio Rad Laboratories, Marne-La-Coquette, France). Children 18 months or older were diagnosed virologically using a TaqMan HIV-1 RNA real-time PCR test with a threshold of 300 copies/mL [35]. Paediatric HIV-1 infection was defined detectable plasma HIV RNA at any age or a HIV-positive serology at age ≥18 months.

All confirmed HIV-infected children attended the programme monthly and had unrestricted and free access to antiretroviral drugs and comprehensive care [36]. Children off and on ART underwent CD4 cell count and CD4 cell percentage measurements every six months. Plasma viral load tests were not routinely available after HIV diagnosis, even in children on ART. Pulmonary X-rays were available for children whose history and symptoms were suggestive of tuberculosis infection. Children initiated ART either at clinical WHO Stage 3 or 4, or at WHO Stage 1 or 2 with impaired immunity defined by age (CD4 count percentage: 25% at <12 months; 20% at 12–35 months; and 15% at ≥36 months) [37]. Cotrimoxazole prophylaxis was given to all HIV-exposed from age six weeks and pursued for all HIV-infected children regardless of their age, as recommended by the national Ivoirian guidelines [36].

In each, centre, members living with HIV from a community-based women's organization provided psychological, social and nutritional support, advice on disclosure, and care for adolescents and orphans. Caregivers were informed about this organization and could contact it at their own initiative. The National Ethics Committee of Côte d'Ivoire approved the Aconda data management system [34].

In the four urban centres of the programme offering paediatric care, paediatric and adult care were offered separately in nearby buildings. Beyond the age of 18 years, young people living with HIV were supposed to be transferred to the adult active file, but if they were reluctant, they could remain in follow-up in the paediatric care centre until the age of 21 years. In 2009, there was neither a formal procedure for this transfer from paediatric to adult care nor specific adolescent care.

In 2009, there was no national guideline about the age at disclosure in HIV-infected children in Côte d'Ivoire, where the median age at initiation of sexual activity is 16 years in women and 18 years in men (Health Demographic Survey, 2005). Strategies of HIV disclosure practices varied according to centres. The accepted standard is that disclosure should be done by the age of 13 years, with the intent of disclosure occurring early enough to prevent secondary sexual transmission.

### Study design

We present data on HIV disclosure in adolescents from the cross-sectional PRADO-CI study conducted in 2009 to explore psychosocial, psychological, behavioural difficulties and their determinants. The PRADO-CI study was nested in the multi-centre prospective paediatric Aconda cohort [33]. All HIV-infected adolescents aged 13–21 years, seen at least once in 2009 in two urban centres (the paediatric ward of the Yopougon Teaching Hospital, and the Centre de Prise en charge de Recherche et de Formation [CePReF]) in Abidjan were eligible for the PRADO-CI study.

### Disclosure process in the study centres

Clinical personnel in the two study centres had similar practices for conducting disclosure. In the Teaching Hospital, two paediatricians and one psychologist were involved, and in the CePReF, two paediatricians, two psychologists, one social worker and one counsellor were involved. Psychologists provided psychological support to the children and the caregivers, not only for HIV disclosure, but also for adherence to ART. In daily practice, psychologists remind the child's caregivers that the disclosure process is an important issue, with an increasing need as the child ages, and help them to inform their children themselves. This issue is discussed among the multidisciplinary staffs.

Clinical personnel assessed disclosure status in routine interviews with the child and his/her caregiver. Disclosure status was documented in patients’ charts, although there was not a standardized format for documentation.

### Data collection and analysis

In each participating paediatric clinic, routine HIV activities were recorded in paper clinical charts and then into an electronic database using unique identification numbers to preserve patient confidentiality.

The following data from patients meeting inclusion criteria were extracted from the paediatric databases:

- Demographic data: date of birth, gender, date of entry in the programme,
- ART regimen: type and date of initiation,
- Cotrimoxazole prophylaxis,
- Clinical condition and CD4 cell count at the time of inclusion.

For the purpose of this study, information in the patient chart regarding adolescent's knowledge of his/her own HIV status (“Is the adolescent informed about his/her HIV status?”) was collected retrospectively from clinic records from entry into HIV care through 2009 using a standardized data extraction form.

Prevalence of disclosure documented in the clinical chart was estimated with 95% confidence intervals (CIs). To compare the characteristics of adolescents according to sites, as well as of their knowledge of their own HIV status, Fisher and Chi-square tests and Kruskal-Wallis tests were used for qualitative variables and quantitative variables, respectively. Correlates of HIV disclosure were investigated using an adjusted logistic regression including all variables associated with disclosure with a *p*-value <0.20 in the univariate analysis. Odds ratio (OR) with 95% CIs were produced. All *p*-values were two-tailed. A *p*-value <0.05 was considered statistically significant. All analyses were performed with SAS software 9.0 (USA).

### Ethics

The PRADO-CI was funded by Sidaction and approved by the National Ethics Committee of Côte d'Ivoire in April 2009. We requested the IRB approval to collect programme data. For this study, no individual informed consent was requested.

## Results

Data from May 2009 to April 2010 from 229 adolescents above 13 years of age (10% of the 2244 children on the database) were included in this analysis. The Yopougon clinic had twice as many adolescents as CePReF. The majority 126 (54.6%) were males and 61.4% were on cotrimoxazole prophylaxis (Table 1). The median age was 15 years (range: 13–21 years). The overall age distribution was: 56.8% were aged 13–15 years, 30.6% were 16–18 years and 12.6% were 19–21 years at the time of the study. A quarter was dual-parent orphans, and half had lost at least one parent. These children were in the HIV care programme for a median of 4.3 years and 93% were on ART for a median of 4.8 years. Their median last CD4 cell count at time of the study was 489/mm^3^ (Table 1).

**Table 1:** Characteristics at study period of all HIV-infected adolescents according to their HIV-care centre (CEPREF and CHU Yopougon) in Abidjan, Côte d'Ivoire, PRADO-CI study, *N=*229

		HIV-care centre		
		
	Overall *N=*229 *n (%)*	CEPREF *n=*79 *n (%)*	YOPOUGON *n=*150 *n (%)*	*p*
Gender				0.279
Female	104 (45.4)	32 (40.5)	72 (48.0)	
Male	125 (54.6)	47 (59.5)	78 (52.0)	
Age group (years)				0.064
[13–15]	130 (56.8)	45 (57.0)	85 (56.7)	
[16–18]	70 (30.6)	19 (24.0)	51 (34.0)	
>18	29 (12.6)	15 (19.0)	14 (9.3)	
History of participation in an HIV research project				0.0001
Yes	135 (60.5)	30 (40.5)	105 (70.5)	
No	88 (39.5)	44 (59.5)	44 (29.5)	
On ART				0.172
Yes	211 (92.9)	70 (89.7)	141 (94.6)	
No	16 (7.1)	8 (10.3)	8 (5.4)	
History of ART regimen change				0.491
Yes	122 (73.1)	34 (69.4)	88 (74.6)	
No	45 (26.9)	15 (30.6)	30 (25.4)	
On cotrimoxazole prophylaxis				0.000
Yes	116 (61.4)	40 (90.9)	76 (52.4)	
No	73 (38.6)	4 (9.1)	69 (47.6)	
Treatment				Not tested
None	3 (1.6)	0 (0.0)	3 (2.1)	
Cotrimoxazole	11 (5.8)	6 (13.6)	5 (3.4)	
ART	70 (37.0)	4 (9.1)	66 (45.5)	
Cotrimoxazole and ART	105 (55.6)	34 (77.3)	71 (48.0)	
Father deceased				0.057
Yes	99 (48.8)	37 (58.7)	62 (44.3)	
No	104 (51.2)	26 (41.3)	78 (55.7)	
Mother deceased				0.113
Yes	120 (56.6)	45 (64.3)	75 (52.3)	
No	92 (43.4)	25 (35.7)	67 (47.7)	
Orphan of both parents				0.344
Yes	55 (25.9)	21 (30.0)	34 (23.9)	
No	157 (74.1)	49 (70.0)	108 (76.1)	
Knowledge of her/his own HIV status				0.003
Yes	63 (27.5)	31 (39.2)	32 (21.3)	
No	130 (56.8)	33 (41.8)	97 (64.7)	
Missing	36 (15.7)	15 (19.0)	21 (14.0)	
	Median (IQR)	Median (IQR)	Median (IQR)	
Length in HIV care in years (*n=*166)	4.3 [4.0–4.6]	4.5 [3.4–4.7]	4.3 [4.3–4.4]	0.249
Length of ART in years (*n=*173)	4.8 [4.0–6.5]	3.9 [2.2–7.1]	5.0 [4.3–6.4]	0.183
Last CD4 cell count in cells/mm^3^ (*n=*221)	489 [251–679]	385 [234–673]	500 [258–680]	0.387

ART=antiretroviral therapy; IQR=interquartile range.

Data on the adolescents’ knowledge of their own HIV status were missing in 36 cases (15.7%), without significant differences in the baseline variables from those for whom data were available. Among the 193 patients who had data regarding knowledge of HIV status available in the chart (84.3%), only 63 (32.6%; 95% CI=26.0–39.3%) had been informed of their HIV status, with no significant gender difference (Table 2). The proportion of adolescents informed increased significantly with age: 18.6% for 13–15 years, 33.3% for 16–18 years and 86.2% for 19–21 years (*p* <0.0001). Adolescents on ART tended to be more likely to be informed of their HIV status (34.5%) than those not treated (13.3%) (*p=*0.11). Those on cotrimoxazole were significantly more likely to be informed of their HIV status (39.6%) than those not receiving contrimoxazole (21.9%) (*p=*0.01). A history of ART regimen change was significantly associated with a higher disclosure rate (*p=*0.003). Adolescents were significantly more often informed of their HIV status in the CePReF (48.4%) compared to the Yopougon Teaching Hospital (24.8%) (*p=*0.001).

**Table 2:** Characteristics at study period associated with the notification of the disclosure of their own HIV status in adolescents in Abidjan, Côte d'Ivoire

	Notification of disclosure in adolescents		Univariate model		Adjusted model	
			
	Yes, *N=*63 *n* (%)	No, *N=*130 *n* (%)	OR [CI 95%]	*p*	aOR [IC 95%]	*p*
Centre						
CEPREF	31 (48.4)	31 (48.4)	2.84 [1.51–5.36]	0.001	3.52 [1.15–10.85]	0.028
CHU Yopougon	32 (24.8)	32 (24.8)	1		1	
Gender						
Female	25 (30.9)	25 (30.9)	0.87 [0.47–1.60]	0.654	1.36 [0.53–3.48]	0.523
Male	38 (33.9)	38 (33.9)	1		1	
Age group (years)						
[13–15]	21 (18.6)	21 (18.6)	1	<0.0001	1	<0.0001
[16–18]	17 (33.3)	17 (33.3)	2.19 [1.03–4.6]		4.21 [1.54–11.49]	
>18	25 (86.2)	25 (86.2)	27.38 [8.61–87.08]		22.08 [5.22–93.49]	
History of participation in a research project						
Yes	44 (37.9)	44 (37.9)	2.05 [1.06–3.96]	0.031	1.93 [0.61–6.08]	0.259
No	17 (23.0)	17 (23.0)	1		1	
On ART						
Yes	61 (34.5)	61 (34.5)	3.42 [0.75–15.63]	0.113	1.15 [0.09–15.21]	0.914
No	2 (13.3)	2 (13.3)	1		1	
On cotrimoxazole prophylaxis						
Yes	40 (39.6)	40 (39.6)	2.34 [1.15–4.78]	0.018	2.04 [0.77–5.41]	0.152
No	14 (21.9)	14 (21.9)	1		1	
History of ART regimen change						
Yes	46 (42.2)	46 (42.2)	4.13 [1.60–10.67]	0.003	3.83 [0.92–15.92]	0.065
No	6 (15.0)	6 (15.0)	1		1	
Orphan of both parents						
Yes	14 (29.2)	34 (70.8)	0.87 [0.61–1.24]	0.438	0.28 [0.09–0.88]	0.290
No	47 (35.3)	86 (64.7)	1		1	

ART=antiretroviral therapy; OR=odds ratio; CI=confidence interval.

Univariate and adjusted logistic regression. PRADO-CI study, *N=*193.

In a fully adjusted model (Table 2), adjusting for centre, gender, age group, history of participation in a research project, ART intake, cotrimoxazole prophylaxis, history of ART regimen change and having no parent, disclosed HIV status remained significantly higher in adolescents followed-up in the CePReF compared to those in the Yopougon Teaching Hospital: adjusted odds ratio (aOR)=3.52 (95% CI: 1.1–10.9; *p*<0.028); and among older adolescents compared to those aged 13–15 years: [16–18 years] aOR=4.2 (95% CI: 1.5–11.5; *p*<0.005) and [>18 years] vs. [13–15 years]: aOR (22.1; 95% CI: 5.2–93.5; *p*<0.0001).

## Discussion

While the disclosure process in adolescents is rarely documented in west Africa, our study provided an opportunity to document the proportion of HIV-infected adolescents informed of their HIV status as known by the healthcare staff in an Ivoirian routine HIV-care context. The reported disclosure rate was low, reaching only 32.6% of the HIV-infected adolescents. In an adjusted model of the correlates of disclosure, this proportion increased significantly with age, with 86.2% of those aged from 19 to 21 years having had disclosure of their status and differed significantly according to the clinical centres.

There are several limitations to our observations. First, the completeness of the data analyzed, collected during outpatient routine follow-up and reported in medical records, might have been the source of bias; we hypothesized that it could have led to an underestimation of the disclosure rate. We observed significantly higher frequency of disclosure notifications in the CePReF centre compared to the Yopougon Teaching Hospital. The CePReF centre had a strong track record in research, had more trained personnel and a smaller adolescent population. The absence of procedures or standardized forms to document HIV disclosure in the medical record may have contributed to a probable underestimation and also to missing data. Second, our study was nested in a cross-sectional study and our documentation was based on a cumulative rate of notification of disclosure, not taking into account the dynamic evolution of the disclosure process. A retrospective qualitative analysis of the history of the disclosure process could allow a deeper exploration of the accuracy and the evolution of the disclosure process; however, this information will remain be limited by memory bias. A prospective cohort to document this dynamic process of disclosure would be desirable. Nevertheless, the disclosure rate from this study may be useful as a baseline reference for HIV-infected adolescents in West Africa.

There are few studies about disclosure to adolescents of their HIV status in west Africa to compare our findings to [14, 23, 27]. Even in settings where HIV prevalence is high, disclosure rate was low with generalized difficulties for caregivers to tell their children about their HIV condition [8, 13, 21, 22, 29, 31, 32]. This low prevalence of disclosure underscores the need for a systematic and a staged approach in disclosing HIV status to infected children in resource-limited countries. In all the previous settings, including our study, the disclosure rate increased with age, but the question of when to inform still remains. As children grow older, their parents/guardians faced the difficult decision of if, when, and how to inform their child of his/her HIV status. As notification of disclosure was higher in children with a history of ART changes, we can hypothesize that it was an opportunity to discuss the reasons of the treatment changes.

Both negative and positive social, psychological and behavioural impacts of disclosure to children have been reported, including improved adherence to medication regimens. While also essential for the secondary prevention of HIV transmission, disclosure may accentuate emotional and behavioural disorders, family conflicts or social stigma perception and may jeopardize confidentiality [6, 38, 39].

Among the factors delaying the disclosure process of HIV status in Africa, caregivers’ knowledge and perceptions could play an important role. In western Kenya, caregivers believed that disclosure might have benefits such as improved ART adherence, especially for older children and better engagement of a helping social network. They also feared, however, that disclosure might have both negative psychological effects for children and negative social effects for their families, including discrimination [31, 32]. In contrast, nearly one-third of caregivers in Democratic Republic of Congo saw no benefits to informing the child of her/his HIV status. A majority of caregivers felt that they themselves were the best to eventually disclose to the child, but some wanted support from healthcare providers [29]. Although we are unable to provide such detailed information in the west-African context, discrimination and fears of a negative psychological impact were reported by both caregivers and staff in the current study as delaying the process of disclosure to children. Disclosure of HIV status to HIV-infected adolescents therefore represents a challenge for the family and for the medical staff [26, 40]. Further investigations are also needed to develop interventions and guide the when and how to disclosure questions without harming adolescents and their families.

Despite emerging evidence of the benefits of disclosure, when and how to disclose the diagnosis of HIV to children remains a clinical dilemma. A further understanding of the HIV-disclosure process and the effect according to the caregivers and their children views is under investigation in the PRADO-CI study using qualitative tools (sociological and psychological interviews and focus group).

## Conclusions

HIV disclosure rate was low among Ivoirian HIV adolescents and site- and age-dependent. There is a need for development of practical interventions to support HIV disclosure to adolescents and provide age-appropriate information about the disease.
